# Adherence to Pre-operative Exercise and the Response to Prehabilitation in Oesophageal Cancer Patients

**DOI:** 10.1007/s11605-020-04561-2

**Published:** 2020-04-20

**Authors:** Laura J. Halliday, Emre Doganay, Venetia Wynter-Blyth, Hayley Osborn, John Buckley, Krishna Moorthy

**Affiliations:** 1 Department of Surgery and Cancer, Imperial College London, London, UK; 2 Oesophago-Gastric Cancer Surgery Unit, St. Mary’s Hospital, Imperial College Healthcare NHS Trust, London, UK; 3 Centre for Active Living, University Centre Shrewsbury/University of Chester, Shrewsbury, Chester, UK

**Keywords:** Oesophageal cancer, Exercise therapy, Pre-operative care, Surgery

## Abstract

**Background:**

Prehabilitation is thought to reduce post-operative respiratory complications by optimising fitness before surgery. This prospective, single-centre study aimed to establish the effect of pre-operative exercise on cardiorespiratory fitness in oesophageal cancer patients and characterise the effect of adherence and weekly physical activity on response to prehabilitation.

**Methods:**

Patients received a personalised, home-based pre-operative exercise programme and self-reported their adherence each week. Cardiorespiratory fitness (pVO_2_max and O_2_ pulse) was assessed at diagnosis, following completion of neoadjuvant chemotherapy (NAC) and immediately before surgery. Study outcomes included changes in fitness and post-operative pneumonia.

**Results:**

Sixty-seven patients with oesophageal cancer underwent prehabilitation followed by surgery between January 2016 and December 2018. Fitness was preserved during NAC and then increased prior to surgery (pV0_2_max Δ = +2.6 ml min^−1^, 95% CI 1.2–4.0 *p* = 0.001; O_2_ pulse Δ = +1.4 ml beat^−1^ 95% CI 0.5–2.3 *p* = 0.001). Patients with higher baseline fitness completed more physical activity. Regression analyses found adherence was associated with improvement in fitness immediately before surgery (*p* = 0.048), and the amount of physical activity completed was associated with the risk of post-operative pneumonia (*p* = 0.035).

**Conclusion:**

Pre-operative exercise can maintain cardiorespiratory fitness during NAC and facilitate an increase in fitness before surgery. Greater exercise volumes were associated with a lower risk of post-operative pneumonia, highlighting the importance progressing exercise programmes throughout prehabilitation. Patients with high baseline fitness completed more physical activity and may require less supervision to reach their exercise goals. Further research is needed to explore stratified approaches to prehabilitation.

**Electronic supplementary material:**

The online version of this article (10.1007/s11605-020-04561-2) contains supplementary material, which is available to authorized users.

## Introduction

Prehabilitation uses the time between diagnosis and surgery to optimise a patient’s functional capacity, which is understood to provide a buffer to withstand the physiological stress of surgery.^1^ There is substantial heterogeneity in the content of prehabilitation programmes, but structured exercise is a major component of most studies.^2^–^5^

Within this rapidly growing area of perioperative medicine, studies have shown that prehabilitation can increase fitness prior to surgery^4^,^6^ and reduce post-operative complications.^7^,^8^ Post-operative pneumonia is a well-acknowledged complication after oesophagectomy and is reported in up to 50% of patients.^9^–^11^ There are multiple reasons for the particularly high rate of post-operative pulmonary complications following an oesophagectomy: intra-operative one lung ventilation, post-operative diaphragm dysfunction and upper abdominal pain limiting coughing and clearing of secretions. A meta-analysis of pre-operative exercise prior to intra-abdominal surgery found it can reduce the incidence of pulmonary complications by 60%.^7^

Unlike rehabilitation, prehabilitation is strictly time constrained. It is therefore important that exercise behaviour is integrated into the pre-operative period efficiently and effectively. Adherence to exercise is challenging in the pre-operative setting.^2^,^4^,^12^ A retrospective review of prehabilitation prior to colorectal cancer surgery reported that 40% of patients did not improve their fitness in response to pre-operative exercise. 6

Neoadjuvant chemotherapy (NAC) has a negative impact on cardiorespiratory fitness, 13,14 and adherence to exercise during this time may be particularly challenging. To date, few studies have examined using pre-operative exercise whilst patients are receiving NAC.^12^,^15^–^17^

The aims of this study are:

1. To examine pre-operative changes in cardiorespiratory fitness during prehabilitation in oesophageal cancer patients receiving NAC
2. To establish whether adherence to a personalised exercise prescription and the amount of physical activity (PA) completed during prehabilitation are related to cardiorespiratory fitness and the incidence of post-operative pneumonia
3. To characterise how baseline fitness affects the amount of PA completed, changes in fitness and the occurrence of post-operative pneumonia

## Materials and Methods

### The PREPARE for Surgery Prehabilitation Programme

PREPARE (physical activity, respiratory exercises, eat well, psychological well being, ask about medications, remove bad habits, enhanced recovery) for Surgery is a personalised, home-based prehabilitation programme for patients with oesophageal cancer. All patients who were diagnosed with resectable disease were invited to participate in the programme, which started immediately after staging investigations and continued during NAC up to the time of surgery (covering a time period of approximately 16 weeks). Results from all patients who completed the programme and underwent surgery from January 2016 to December 2018 were included in this study.

A home-based exercise programme is agreed with the patient. In keeping with WHO guidelines, patients were prescribed a personalised exercise programme with the aim of trying to achieve a minimum of 600 MET minutes week^−1^ of aerobic exercise,^18^ which equates to 150 min of moderate/vigorous-intensity activity. Strength exercises were also prescribed alongside aerobic activities. The programme was personalised according to FITT principles (frequency, intensity, time and type)^19^ using the results of submaximal exercise testing and information on activities of daily living, previous exercise behaviour, medical co-morbidities and social circumstances (see Appendix 1 for an example of a personalised exercise programme).

Patients were trained to self-monitor and self-regulate exercise intensity using the Borg scale rating of perceived exertion (RPE) 20. A weekly telephone touch-point was held with an exercise therapist. Providing the goals were achieved, the exercise programme was progressed in frequency, time and then intensity, according to FITT principles, with the aim achieving a volume of ≥ 1200 MET minutes week^−1^ or more per week (300 min of moderate/vigorous-intensity activity).

For those who were unable to meet their goals each week, the programme was adapted to their current condition and re-evaluated at the next weekly touch-point. Changes were made to the frequency of each exercise, followed by the duration and then type of exercises if further modification was required. Patients also received consultations with clinical nurse specialists if needed. During these sessions, the rationale for prehabilitation was reinforced, potential barriers and facilitators to exercise were explored and motivational interviewing techniques were used to facilitate positive behaviour change. 21

Following surgery, an enhanced recovery protocol (ERP) was used, with predefined targets for extubation, mobilisation, oral intake, removal of drains and nasogastric tubes and pain control (see Appendix 2). All patients who underwent PREPARE for Surgery followed the same post-operative protocol.

### Measurement of Cardiorespiratory Fitness

Submaximal exercise testing using the Chester Step Test (CST) was used to assess the predicted maximal oxygen uptake (pVO_2_max) and O_2_ pulse.^22^,^23^ Submaximal exercise testing has been used in previous clinical studies and is appropriate for prescription of home-based exercises.^24^,^25^ The CST was performed by a trained exercise therapist. During the test, patients walked up and down a step at a predefined frequency using a metronome. Three different step heights were used dependent on the patient’s abilities. Every 2 min, the speed of the metronome was increased. The test finished when the patient felt unable to continue, was unable to maintain the metronome speed or reached over 70% of their heart rate reserve.

The CST is a validated method to estimate aerobic power and maximal oxygen uptake (pVO_2_max).^23^,^26^ From the VO_2_ calculated during the CST, O_2_ pulse was derived. This an indirect representation of stroke volume and demonstrates the central cardiac contribution to overall oxygen uptake.^27^

The CST was undertaken at three time points during prehabilitation:

- PREPARE 1 (P1) = diagnosis (baseline)
- PREPARE 2 (P2) = completion of NAC. For patients who did not receive NAC this was their baseline measurement (4 to 6 weeks prior to surgery)
- PREPARE 3 (P3) = 1 week prior to surgery

In respect of the known estimate errors of the CST and effects of test-retest familiarity,^23^,^26^,^28^ and based on studies examining the clinical effects of improving pVO_2_max,^29^ an improvement in fitness was defined as an increase of 10% or more in pVO_2_max.

### Measurement of Adherence

Patients self-reported the frequency, duration and intensity with which they completed each exercise every week using exercise diaries (see Appendix 1). The recorded RPE scores were used to derive the percentage of VO_2_max and METSmax at which they exercised, 30 and thus, using the METSmax derived from the CST, the estimated achieved intensity in METS was calculated. The weekly exercise duration, intensity and frequency for each activity were multiplied to quantify the volume of physical activity (PA) in MET minutes week^−1^. Weekly adherence was calculated as a percentage of actual/prescribed MET minutes week^−1^.

There is no standardised method for measuring adherence or defining acceptable levels of adherence in exercise studies. 31 We therefore pragmatically defined high adherence as an average weekly adherence of 75% or greater.

### Definition of Pneumonia

The diagnosis of post-operative pneumonia was defined according to the CDC definition for the clinical diagnosis of a hospital-acquired pneumonia (see Appendix 3).^32^

### Statistical Analysis

Changes in pVO_2_max and O_2_ pulse during prehabilitation were assessed using a repeated-measures one-way analysis of variance (ANOVA) for patients who underwent NAC and had three measurements, and a paired *T* test for those who did not have NAC and therefore had two measurements. If significant difference was determined with the ANOVA, post hoc paired *T* tests with a Bonferroni correction were used to determine where the paired differences occurred.

Multivariate analyses of the factors associated with improvement in fitness and with post-operative pneumonia were performed using binary logistic regression. Chi-squared tests were used to establish the effects of an increase in fitness and high adherence on the rate of post-operative pneumonia. Student’s *T* tests were used to compare baseline fitness in patients with high adherence to the rest of the cohort and to compare baseline fitness according to changes in fitness during prehabilitation. To assess the relationship between baseline fitness and average weekly PA, a Pearson correlation test was used. Finally, to assess the variability of self-reported exercise adherence, intra and inter-person coefficient of variances were calculated.

Two-tailed tests were used throughout with a significance level of *p* < 0.05. Statistical analysis was performed using SPSS version 25 (IBM, New York, USA).

## Results

Between January 2016 and December 2018, 79 patients with oesophageal or gastro-oesophageal junctional adenocarcinoma entered the PREPARE for Surgery programme. Ten patients did not complete the programme due to a change in clinical status precluding resection (disease progression, development of metastases or deterioration in medical co-morbidities). One patient declined to participate in the programme, and one patient declined surgery after starting treatment. The remaining 67 patients who completed the programme and underwent surgery were included in this study. Characteristics for these patients are shown in Table 1. Of the 67 patients, 60 underwent NAC and had three pre-operative assessments of fitness (P1, P2 and P3). The remaining seven patients did not receive NAC and therefore had two pre-operative assessments (P2 and P3).

**Table 1:** Study participant characteristics

Characteristics	
Age—mean (S.D.)	66 (9.7)
ASA grade—*n* (%) ASA 1	0
ASA 2	57 (85%)
ASA 3	10 (15%)
ASA 4	0
Charlson Comorbidity Index score—mean (range)	5 (2–8)
Pre-operative T stage—*n* (%) T1	5 (8%)
T2	11 (16%)
T3	44 (66%)
T4	7 (10%)
Pre-operative N stage – n (%) N0	23 (34%)
N1	33 (50%)
N2	8 (12%)
N3	3 (4%)
NAC—*n* (%)	60 (90%)
Baseline weight—mean kg (S.D.)	79.7 (19.4)
Baseline pVO_2_max—mean (S.D.)	23.8 (6.4)
Adherence—mean % (S.D)	
Overall	64% (30.1)
P1–2	56% (30.1)
P2–P3 NAC patients	65% (36.3)
P2–3 non-NAC patients	85% (22.5)
Post-operative pneumonia—*n* (%)	22 (33%)
Overall complications—*n* (%)	44 (66%)
Length of stay—median days (IQR)	10 (8–16)

*S.D.* standard deviation, *IQR* interquartile range

Eleven patients had a three-stage oesophagectomy and 56 had a two-stage procedure. Both the abdominal and thoracic components were performed as open procedures in 62 patients (93%); five patients had a laparoscopic abdominal phase followed by an open thoracotomy.

### Physical Activity and Adherence

The overall mean PA was 989 MET minutes week^−1^ (S.D. 805); between P1 and P2, this was 848 MET minutes week^−1^ (S.D 667), and this rose to 1228 MET minutes week^−1^ (S.D 1236) between P2 and P3. Coefficient of variance for self-reported adherence within individual patients was 49% (intra-person variability) and between patients was 38% (inter-person variability).

### Changes in Fitness during Prehabilitation

In patients who received NAC, both pVO_2_max and O_2_ pulse changed significantly during prehabilitation (Tables 2 and 3 respectively). pVO_2_max and O_2_ pulse were preserved between P1 and P2, followed by a significant increase between P2 and P3: Δ pV0_2_max = + 2.6 ml min^−1^ kg^−1^ (95% CI 1.2–4.0); ΔO_2_ pulse = + 1.4 ml beat^−1^ (95% CI 0.5–2.3).

**Table 2:** pVO_2_max during prehabilitation

	pVO2_2_max at P1 (ml min^−1^ kg^−1^)	pVO2_2_max at P2 (ml min^−1^ kg^−1^)	pVO2_2_max at P3 (ml min^−1^ kg^−1^)	*p* value
Patients receiving neoadjuvant therapy				
	24.3 (22.5–26.2; S.D. 6.5)	23.2 (21.4–25.0; S.D. 6.3)	25.8 (24.0–27.5; S.D. 6.1)	0.01
Post hoc analysis	24.3 (22.5–26.2)	23.2 (21.4–25.0)		0.292
		23.2 (21.4–25.0)	25.8 (24.0–27.5)	0.001
Whole cohort				
	n/a	23.0 (21.3–24.6; S.D. 6.3)	25.9 (24.3–27.5; S.D. 6.2)	0.001

Results displayed as mean (95% confidence interval; S.D.)

*n/a* not applicable, *S.D.* standard deviation

**Table 3:** O_2_ pulse during prehabilitation

	O_2_ pulse at P1 (ml beat^−1^)	O_2_ pulse at P2 (ml beat^−1^)	O_2_ pulse at P3 (ml beat^−1^)	*p* value
Patients receiving neoadjuvant therapy				
	14.7 (13.7–15.7; S.D. 3.3)	14.1 (13.0–15.1; S.D. 3.4)	15.5 (14.4–16.5; S.D. 3.5)	0.002
Post hoc *analysis*	14.7 (13.7–15.7)	14.1 (13.0–15.1)		0.264
		14.1 (13.0–15.1)	15.5 (14.4–16.5)	0.001
Whole cohort				
	n/a	13.9 (13.30–14.8; S.D. 3.3)	15.4 (14.5–16.3S.D.3.4)	0.001

Results displayed as mean (95% confidence interval; S.D.)

*n/a* not applicable, *S.D.* standard deviation

In the whole cohort (including those who did not undergo NAC), both pVO_2_max and O_2_ pulse increased significantly between P2 and P3: Δ pVO_2_max = + 3.0 ml min^−1^ kg^−1^ (95% CI = 2.0–4.0); ΔO_2_ pulse = + 1.6 ml beat^−1^ (95% CI = 0.9–2.2).

### Factors Predictive of Improvement in Cardiorespiratory Fitness Between P1 and P2

Twenty-one per cent of patients increased their pVO_2_max by 10% or more between P1 and P2. There was no significant relationship between the increase in fitness between P1 and P2 and age, pre-operative stage, baseline fitness, adherence or average weekly PA completed between P1 and P2 (*p* > 0.05).

### Factors Predictive of Improvement in Cardiorespiratory Fitness Between P2 and P3

Fifty-two per cent of patients increased their pVO_2_max by 10% or more between P2 and P3. Adherence with prescribed exercise was significantly associated with the chance of increasing pVO_2_max between P2 and P3 (Table 4; *p* = 0.048). There was no significant effect of age, the use of NAC, pre-operative stage, baseline fitness or average weekly PA completed between P2 and P3.

**Table 4:** Factors associated with improvement in pVO_2_max between P2 and P3

Variable	Odds ratio (95% CI)	B	SE	*p* value
NAC	0.16 (0.00 to 2.28)	− 4.14	2.54	0.999
Age	0.98 (0.88 to 1.09)	− 0.18	0.56	0.752
Charlson comorbidity index	1.63 (0.67 to 4.02)	0.49	0.46	0.286
Clinical stage (1)	0.15 (0.00 to 40.96)	− 1.89	27.7	0.999
Clinical stage (2)	0.10 (0.01 to 1.37)	− 2.34	1.36	0.840
Clinical stage (3)	0.49 (0.84 to 2.82)	− 0.72	0.90	0.423
Baseline pVO2max (ml min^−1^ kg^−1^)	0.99 (0.88 to 1.10)	− 0.15	0.59	0.795
Total adherence (%)	49.38 (1.04 to 2356.0)	3.90	1.97	0.048
Average PA (MET min week^−1^)	0.99 (0.99 to 1.00)	− 0.002	0.001	0.126

### Factors Predictive of Post-Operative Pneumonia

Baseline characteristics, adherence, PA, changes in fitness and ERP compliance for patients who developed post-operative pneumonia are shown in Table 5. Average weekly PA over the whole programme was significantly associated with the risk of post-operative pneumonia (Table 6; *p* = 0.035). There was no significant effect of age, the use of NAC, pre-operative stage, baseline fitness or adherence to prescribed exercise.

**Table 5:** Characteristics of patients who did and did not develop post-operative pneumonia

Characteristics	Patients who developed pneumonia	Patients who did not develop pneumonia	*p* value
*N* (%)	22 (33%)	45 (67%)	
Age—mean (S.D.)	68.4 (10.0)	65.3 (9.4)	0.214
Pre-operative T stage—*n* (%)			0.725
T1	2 (9%)	3 (7%)	
T2	3 (14%)	9 (20%)	
T3	15 (68%)	28 (62%)	
T4	2 (9%)	5 (11%)	
Pre-operative N stage—*n* (%)			0.494
N0	5 (22%)	18 (40%)	
N1	13 (59%)	19 (43%)	
N2	3 (14%)	6 (13%)	
N3	1 (5%)	2 (4%)	
NAC—*n* (%)	19 (86%)	41 (91%)	0.551
Baseline pVO_2_max—mean (S.D.)	23.9 (5.4)	23.7 (6.8)	0.911
Change in pVO_2_max—mean (S.D.)			
P1–2	−9.9 (16.7)	3.3 (27.6)	0.068
P2–P3	18.1 (22.8)	14.1 (17.8)	0.459
Adherence—mean % (S.D.)			
Overall	62% (26.3)	65% (32.0)	0.807
P1–2	55% (34.0)	57% (22.2)	0.790
P2–P3	67% (36.7)	69% (32.6)	0.813
Weekly PA—mean MET min week^−1^ (S.D.)			
Overall	785 (528)	1084 (895)	0.184
P1–2	653 (349)	960 (779)	0.157
P2-P3	954 (699)	1332 (1381)	0.334
Laparoscopic abdominal phase—*n* (%)	2 (9%)	3 (7%)	0.723
Compliance with enhanced recovery protocol—*n* (%)			
Mobilization	4 (18%)	19 (42%)	0.094
Nasogastric tube removal	7 (32%)	31 (69%)	0.006
Drain removal	5 (23%)	27 (60%)	0.006
Oral intake	1 (5%)	24 (53%)	0.001
Pain control	11 (50%)	27 (60%)	0.492
Day 0 extubation	14 (64%)	33 (73%)	0.577

**Table 6:** Factors associated with development of post-operative pneumonia

Variable	Odds ratio (95% CI)	B	SE	*p* value
NAC	0.21 (0.01 to 28.32)	− 1.57	2.51	0.531
Age	1.03 (0.92 to 1.15)	0.31	0.57	0.588
Charlson comorbidity index	1.35 (0.62 to 2.91)	0.30	0.39	0.449
Clinical stage (1)	3.11 (0.13 to 734.1)	1.14	2.79	0.684
Clinical stage (2)	0.20 (0.13 to 3.28)	− 1.59	1.42	0.262
Clinical stage (3)	0.87 (0.14 to 5.33)	− 1.42	0.93	0.878
Baseline pVO2max	1.11 (0.99 to 1.25)	0.10	0.61	0.083
Total adherence (%)	24.23 (0.82 to 715.6)	3.19	1.73	0.065
Average PA (MET min week^−1^)	0.99 (0.95 to 0.99)	− 0.02	0.01	0.035

**Fig. 1:**
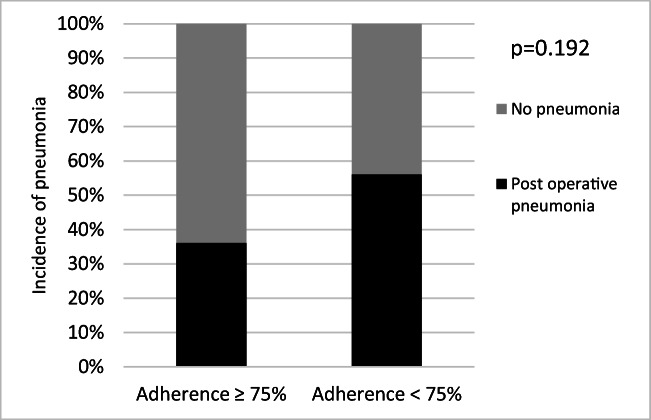
Incidence of post-operative pneumonia in patients with high adherence

**Fig. 2:**
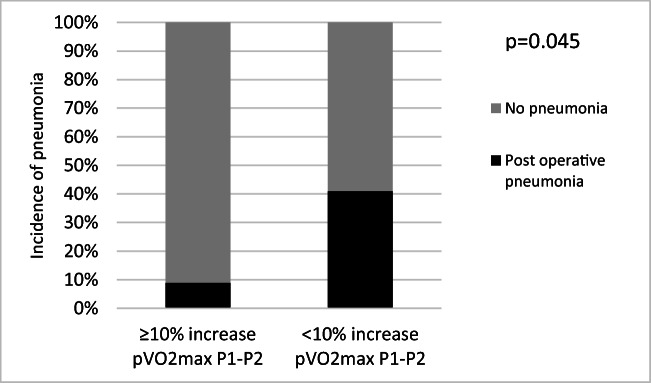
Incidence of post-operative pneumonia in patients who increased pVO_2_max during chemotherapy

Patients with ≥ 75% adherence had a lower incidence of post-operative pneumonia, although this difference did not reach statistical significance (22% vs 39%, *p* = 0.192; Fig. 1). Patients who increased pVO_2_max between P1 and P2 had a lower incidence of post-operative pneumonia than patients who either maintained or had a fall in pVO_2_max (9% vs 41%, *p* = 0.045; Fig. 2). There was no significant difference in the incidence of pneumonia between patients who increased their pVO_2_max between P2 and P3 and those who did not (32% vs 34%, *p* = 0.855).

### Baseline Fitness and Adherence

**Fig. 3:**
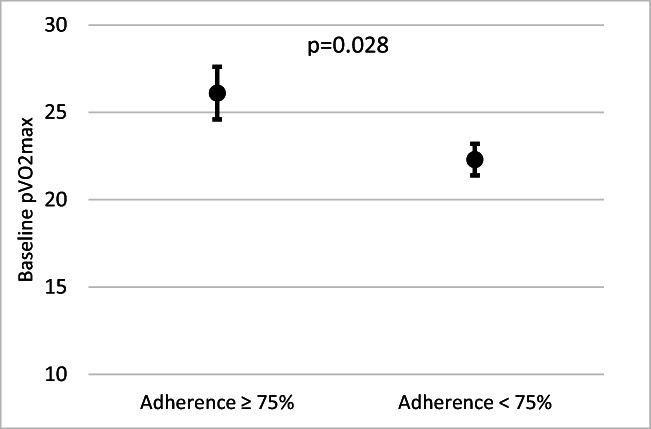
Baseline fitness in patients with high adherence. Results displayed as mean (± SE)

Patients who had ≥ 75% adherence had a higher baseline pVO_2_max than those with less than 75% adherence (26.1 vs 22.3, *p* = 0.028; Fig. 3). There was also a moderate correlation between baseline pVO_2_max and average weekly PA completed throughout the programme, with higher levels of PA in patients with a higher baseline fitness (r = 0.340, *p* = 0.008).

### Baseline Fitness and Changes in Fitness

**Fig. 4:**
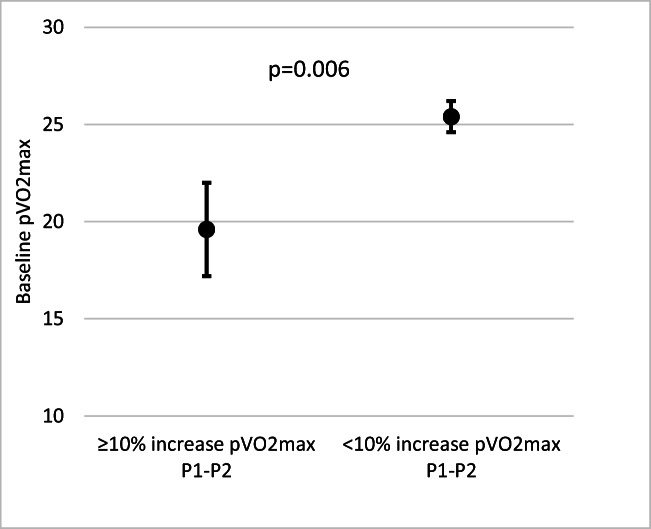
Baseline fitness in patients who increased their fitness. Results displayed as mean (± SE)

Patients who increased their pV0_2_max between P1 and P2 had a lower baseline fitness than those who either maintained or had a fall in pVO_2_max during this time (19.6 vs 25.4, *p* = 0.006; Fig. 4). Baseline pVO_2_max was comparable between patients who did and did not increase their fitness between P2 and P3 (23.5 vs 24.4, *p* = 0.620).

### Baseline Fitness and Pneumonia

There was no significant difference in baseline pVO_2_max between patients who did and did not develop a post-operative pneumonia (23.9 vs 23.7, *p* = 0.903).

## Discussion

In this 3-year retrospective analysis of patients with oesophageal cancer, we have demonstrated that with prehabilitation, cardiorespiratory fitness can be maintained during NAC and this is then followed by an increase in fitness prior to surgery. Whilst a recovery in fitness following NAC could be anticipated, without prehabilitation the patient is recovering after a substantial fall in fitness from their baseline.^13^,^14^ We have seen that starting prehabilitation once the decision for curative surgery is made can counteract the effects of chemotherapy and facilitate an increase in exercise intensity just prior to surgery.

Oesophageal cancer surgery has a high post-operative morbidity; the overall incidence of complications often exceeds 70% and post-operative pneumonia rates of up to 50% are reported.^9^–^11^,^33^ In this study, we have demonstrated that the amount of PA completed was significantly associated with the risk of pneumonia, with the incidence reducing as the volume of PA in MET min week^−1^ increases. Patients should be encouraged to be as active as possible, with review and escalation of programmes to increase PA throughout prehabilitation.

Adherence to the personalised exercise programme was related to improvements in fitness in the period immediately prior to surgery. We found a mean increase in pVO_2_max of 2.6 ml min^−1^ kg^−1^ after NAC and 2.9 ml min^−1^ kg^−1^ amongst the whole cohort, which is comparable to changes seen in other prehabilitation studies.^34^ Changes of this magnitude are associated with benefits in other clinical situations.^29^,^35^

Reported rates of adherence to prehabilitation exercise programmes vary from 16 to 97%,^4^ with an average of 70% adherence in home-based programmes.^36^ To date, there is no standardised way to assess adherence to exercise. We have calculated a weekly adherence percentage for each patient, which not only assesses frequency and/or time spent exercising but includes the relative intensity of the exercise and therefore gives a more detailed reflection of the true exercise volume.^31^

NAC had a negative impact on adherence, with an average adherence of 56%. Following NAC, average adherence increased to 65% but remained lower than the 85% seen in patients who did not receive NAC. The adherence percentage does not reflect the dose of exercise, as the amount of PA prescribed varied from patient-to-patient. Whilst adherence was challenging for patients during NAC, the average amount of PA achieved exceeded the WHO recommendations of 600 MET minutes week^−1^.^18^ In the 4- to 6-week period before surgery, the average weekly PA level increased by over 40% to more than twice the WHO target. However, further research is still needed to better understand and evaluate ways to support and encourage behaviour change, especially during NAC.

The effect of prehabilitation upon compliance with ERPs is not known. We found low levels of ERP compliance in our patient population and for half of the ERP elements, the compliance was significantly lower in patients who developed post-operative pneumonia. This is unsurprising as poor ERP compliance may be both a consequence of post-operative complications, as well as a risk factor. ERPs should be seen as a continuum of prehabilitation, and efforts to integrate the two pathways may have a positive impact on ERP compliance.

Patients with a low baseline fitness were more likely to increase their fitness during NAC. In parallel to this, those with higher baseline fitness completed more PA and had higher adherence to exercise during prehabilitation. These patients may require less supervision and support to reach their exercise and fitness goals. This raises the possibility of a stratified approach to prehabilitation; as well as a personalised exercise prescription, the level of supervision could be tailored to the patient. In centralised services, such as oesophagogastric cancer, this could have logistical and financial benefits for both the patient and the healthcare provider by minimising disruption for patients who can undertake prehabilitation independently and generating a more efficient use of resources to support patients who need more supervision to achieve their exercise goals.

There are a number of limitations to this study. It is an observational study of patients undergoing surgery in a single centre. Adherence is based on self-reported exercise behaviour. We saw high inter-patient and intra-patient variability of reported exercise levels, demonstrating that patients were reporting different amounts of PA each week, and this suggests that they were actively engaged in the process of self-reporting. However, it is a subjective measurement and the accuracy of these reports cannot be verified. To account for the weekly variation in the amount of PA, the average weekly PA was used in our data analysis.

The intensity of exercise was self-reported using RPE. Although this is a validated scale with which to assess the percentage of METSmax,^30^,^37^,^38^ it may be subject to self-reported measurement bias and therefore may overestimate or underestimate the intensity that was reported. The use of heart rate monitors and activity trackers during exercise may provide a more accurate way to assess intensity and exercise volume, and this should be investigated further. More research is also needed to explore which patients benefit most from prehabilitation and how programmes could be stratified, including identifying criteria to decide the level of support and supervision for each patient.

## Conclusion

The time available to improve a patient’s fitness prior to surgery is tightly limited, and therefore, it is important to use this time efficiently. We have shown that sustained levels of PA can be achieved during NAC, and this can protect against the impact of chemotherapy on cardiorespiratory fitness. Increasing amounts of PA completed during prehabilitation was associated with a lower risk of post-operative pneumonia. Therefore, we propose that exercise goals should not be seen as a fixed concept during prehabilitation; instead, a personalised approach should encourage patients to continually escalate and maximise their PA to achieve the maximum benefit in the pre-operative period. Patients with higher baseline fitness completed more PA and had higher adherence. Further research is needed to establish which patients benefit most and thus identify potential screening criteria for a stratified approach to the delivery of prehabilitation programmes.

## Electronic supplementary material

ESM 1 (DOCX 16.8 KB)

ESM 2 (DOCX 39.9 KB)

ESM 3 (DOCX 14.2 KB)
