# Supplementary material for: Adherence to Pre-operative Exercise and the Response to Prehabilitation in Oesophageal Cancer Patients
Source: J Gastrointest Surg. 2020 Apr 20;25(4):890–9. doi: 10.1007/s11605-020-04561-2 (PMC8007503; doi:10.1007/s11605-020-04561-2)
Supplement: Supplementary file 1 — (DOCX 16.8 KB) [file 11605_2020_4561_MOESM1_ESM.docx]

**exercise prescription card**

**Name: Week Number: Date at start of the week:**

| **Activity** |  | **Monday** | **Tuesday** | **Wednesday** | **Thursday** | **Friday** | **Saturday** | **Sunday** |
| --- | --- | --- | --- | --- | --- | --- | --- | --- |
| **Bike**  15mins, 5x a week  RPE 14 | Duration |  |  |  |  |  |  |  |
|  | Intensity RPE |  |  |  |  |  |  |  |
| **Walking**  30mins, 5x a week  RPE 14 | Duration |  |  |  |  |  |  |  |
|  | Intensity RPE |  |  |  |  |  |  |  |
| **Seat to stand**  2x 15 reps, 5x a week | Duration |  |  |  |  |  |  |  |
|  | Intensity RPE |  |  |  |  |  |  |  |
| **Squat**  3x 15 reps, 5x a week | Duration |  |  |  |  |  |  |  |
|  | Intensity RPE |  |  |  |  |  |  |  |
| **Stair climbing**  6 mins, 5x a week | Duration |  |  |  |  |  |  |  |
|  | Intensity RPE |  |  |  |  |  |  |  |
| **Press ups**  3 x 15 reps, 5x a week | Duration |  |  |  |  |  |  |  |
|  | Intensity RPE |  |  |  |  |  |  |  |
