# Supplementary material for: Adherence to Pre-operative Exercise and the Response to Prehabilitation in Oesophageal Cancer Patients
Source: J Gastrointest Surg. 2020 Apr 20;25(4):890–9. doi: 10.1007/s11605-020-04561-2 (PMC8007503; doi:10.1007/s11605-020-04561-2)
Supplement: Supplementary file 2 — (DOCX 39.9 KB) [file 11605_2020_4561_MOESM2_ESM.docx]

Enhanced recovery protocol

*POD = post-operative day; NG = naso-gastric*
