# Supplementary material for: Adherence to Pre-operative Exercise and the Response to Prehabilitation in Oesophageal Cancer Patients
Source: J Gastrointest Surg. 2020 Apr 20;25(4):890–9. doi: 10.1007/s11605-020-04561-2 (PMC8007503; doi:10.1007/s11605-020-04561-2)
Supplement: Supplementary file 3 — (DOCX 14.2 KB) [file 11605_2020_4561_MOESM3_ESM.docx]

**Center for Disease Control and Prevention Surveillance Definition for Clinical Diagnosis of Hospital Acquired Pneumonia [23]**

**Radiological signs**

≥2 serial radiographs with at least one of the following:

- New or progressive and persistent infiltrate
- Consolidation
- Cavitation

**Clinical signs**

At least one of the following:

- Fever (temperature >38°C) with no other recognised cause
- Leucopenia (<4.0 x 10^9^ cells/L) or leucocytosis (>12.0 x 10^9^ cells/L)
- For adults ≥70 years of age, altered mental state with no other recognised cause

And at least two of the following:

- New onset of purulent sputum, change in character of sputum, increased respiratory secretions or increased suctioning requirements
- New-onset of worsening cough, or dyspnoea, or tachypnoea
- Rales or bronchial breath sounds
- Worsening gas exchange (PaO2:FiO2 ratio ≤240, increased oxygen requirement, increased ventilation demand)
